# Challenges of DMEK Technique with Young Corneal Donors’ Grafts: Surgical Keys for Success—A Pilot Study

**DOI:** 10.3390/jcm12196316

**Published:** 2023-09-30

**Authors:** Mayte Ariño-Gutierrez, Mercedes Molero-Senosiain, Barbara Burgos-Blasco, Beatriz Vidal-Villegas, Pedro Arriola-Villalobos, Jose Antonio Gegundez-Fernandez, Gregory Moloney, Luis Daniel Holguín

**Affiliations:** 1 Department of Ophthalmology, Hospital Clínico San Carlos, Instituto de Investigación Sanitaria (IdISSC), 28040 Madrid, Spain; 2 Tissue Bank, Hospital Clínico San Carlos, 28040 Madrid, Spain; 3 Department of Ophthalmology and Visual Sciences, University of British Columbia, Vancouver, BC V6T 1Z4, Canada; 4 Unidad de Segmento Anterior, Centro Oftalmológico Oftalmosanitas, Bogotá 110741, Colombia

**Keywords:** Descemet Membrane Endothelial Keratoplasty, corneal transplantation, eye banking, young donor

## Abstract

Purpose: To report on the surgical maneuvers recommended for a successful unfolding of very young donors in order to accomplish an uneventful Descemet Membrane Endothelial Keratoplasty (DMEK) surgery. Methods: Five patients (three females and two males, mean age 71.2 ± 6.7 years) with Fuchs endothelial cell dystrophy who underwent DMEK with very young donors (between 20 and 30 years old) were included. The following demographic data were assessed: donor’s age, donor’s endothelial cell density (ECD), preservation time, recipient’s age and sex and unfolding surgical time. Best-corrected visual acuity (BCVA; decimal system), ECD and corneal central thickness (CCT) were assessed preoperatively and at 6-month follow-up. Results: Donors’ mean age was 23.6 ± 3.6 years (range 21 to 30) and the mean ECD was 2748.6 ± 162.6 cells/mm^2^. All of them underwent an uneventful DMEK as a single procedure performed by one experienced surgeon (MAG) with a mean unfolding time of 7.2 ± 4.9 min (range 4 to 15). The essential steps, including patient preparation as well as DMEK graft implantation, orientation, unrolling and centering are detailed. At 6 months, BCVA was 0.6 ± 0.2, ECD was 1945.0 ± 455.5 cells/mm^2^ and CCT was 497.0 ± 19.7 microns. Conclusions: We hereby present the keys to overcome tightly scrolled grafts of very young donors, which prove perfectly suitable for DMEK surgery. The graft shape tends towards a double-roll and specific maneuvers are strongly recommended.

## 1. Introduction

Descemet Membrane Endothelial Keratoplasty (DMEK) has become the most popular type of corneal transplant since Melles et al. described it in 2006. It allows for decreased rates of graft rejection, better visual acuity outcomes and faster recovery compared to Descemet stripping automated endothelial keratoplasty (DSAEK) [1]. However, inherent challenges to this surgery, such as graft preparation, anterior chamber (AC) unfolding and graft adherence to the posterior stroma, make DMEK a technically demanding procedure that requires significant training and experience to master [2].

The number of DMEK surgeries is growing worldwide as it has become the surgery of choice for corneal endothelial diseases, which has resulted in an increasing demand for tissue. In this regard, theoretically, accepting younger donors with better endothelial cell density (ECD) would be preferable for longer expected graft survivals and would increase the availability of tissue. Over the last two decades, corneal donor characteristics have been extensively studied. Postoperative results of young (e.g., under 50 years of age) and older (e.g., above 50 years of age) donors have been compared considering preoperative ECD, preservation time, death-to-preservation time and donor diabetes status, but no differences in 6-month ECD or rebubbling rates have been found [3]. Schaub et al. compared young (ages ranging from 17 to 40 years old) and old donors’ surgical results. Postoperative best corrected visual acuity (BCVA), ECD, corneal thickness, graft biomechanical behaviour and rebubbling rates were comparable between both groups at 6 and 12 months [2].

Although younger donors have been described as providing safe tissue for DMEK surgery, they represent a surgical challenge as their grafts tend to result in tighter rolls with a longer intraoperative unfolding time. There is still limited data available on the outcomes and complication rates following the use of younger donor grafts. Few techniques have specifically addressed tighter rolls in the literature, but none have described the particular behaviour of twenty-year-old donors’ DMEK grafts [4,5].

The purpose of this paper is to report on the surgical maneuvers recommended for a successful unfolding of very young donors in order to accomplish an uneventful DMEK surgery. To the best of our knowledge, this is the first paper describing a specific surgical technique with this kind of young donors’ corneal grafts.

## 2. Materials and Methods

In this case series, we present the patients’ characteristics and the DMEK surgical techniques on five very young corneal donor endothelial grafts performed at Hospital Clinico San Carlos in Madrid, Spain. The paper was conducted in compliance with the tenets of the Declaration of Helsinki. No Ethics Committee approval was required.

The five eligible donor corneas were from healthy donors between the ages of 20 and 30 years old and were preserved in Eusol-C preservation media (Corneal Chamber, Alchimia, Ponte San Nicolo, Italy) at 4 °C for 3 to 4 days after enucleation. Corneal donor characteristics were analysed with EKA-10 (Konan, Japan).

The following demographic data were assessed: donor’s age, donor’s ECD, preservation time, recipient’s age and sex and unfolding surgical time (from the moment after injection until the gas bubble was inserted under the graft). BCVA (decimal system), ECD and corneal central thickness (CCT) were also evaluated preoperatively with a 6-month follow-up in all cases. Cases where a 12-month follow-up was available were also registered.

### Surgical Technique

All patients underwent DMEK surgery under general anesthesia (Video S1). Prior to surgery, an iridotomy at 6 o’clock was performed with a neodymium-doped ytrium-aluminum-garnet (Nd:YAG) laser to prevent postoperative angle block and secondary iris ischaemia. This was carried out in the clinic the day the patient was listed for surgery.

Descemet membrane graft dissection of the young donor corneas was performed with a modified SCUBA technique using blunt dissection and starting at the scleral spur. No difficulties were found in any of the cases, such as tight adhesions or radial tears. Three donor corneas were split for Deep Anterior Lamellar Keratoplasty (DALK) and DMEK surgeries, so the endothelial graft was positioned on a bandage contact lens (Purevision, Balafilcon A, Baush & Lomb, Ontario, Canada) and punched as previously described by Melles’ group [6].

**Figure 1:**
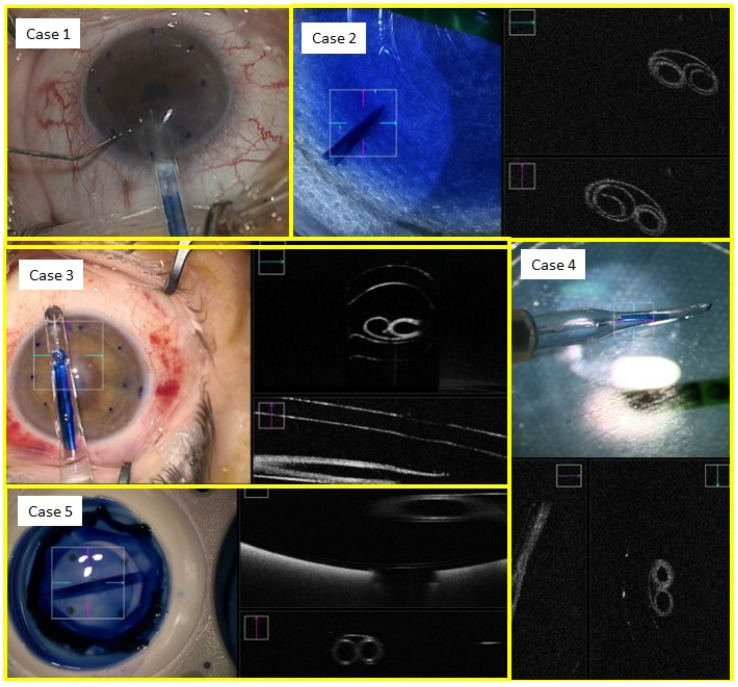
Five cases of tight double-roll configuration DMEK graft.

Once the graft was free in all 360 degrees, it was stained with trypan blue 0.06% (VisionBlue; DORC International, Zuid-Holland, Netherlands) for 2 min. Given the availability of intraoperative optical coherence tomography (OCT), the graft was not marked. In all 5 cases, the tissue rolled into a perfect double-roll without the need for Balanced Salt Solution (BSS) bursts (Figure 1). The graft was used immediately after staining.

Preparing the host cornea was similar to any other DMEK procedures. There was no need to remove the epithelium in any of the patients. Three incisions were made—the main wound with a 2.75 mm keratome at 12 o’clock and two side ports at 2 and 10 o’clock with a 23 G MVR blade. The descemetorhexis was performed with a reverse Sinskey hook under air. A 2 mm peripheral rim of host Descemet was left to avoid peripheral corneal oedema and bullae postoperatively.

**Figure 2:**
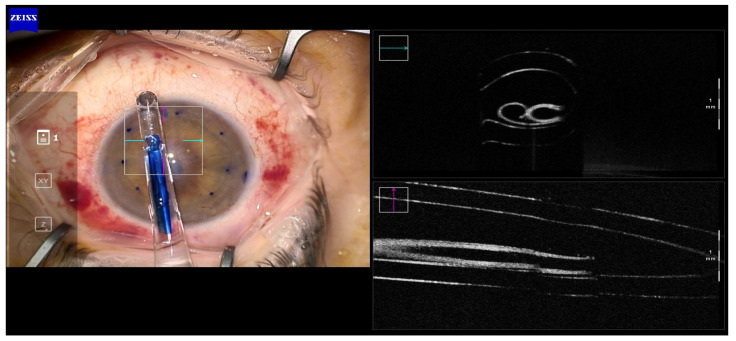
Case 3. Tight double-roll in the right orientation before injecting the graft in anterior chamber. (Callisto, Zeiss Meditec, Jena, Germany).

**Figure 3:**
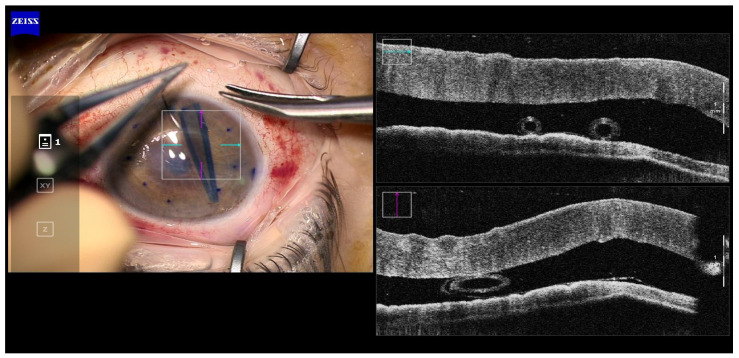
Case 2. Double-roll in the right orientation immediately after being injected in the anterior chamber. (Callisto, Zeiss Meditec, Jena, Germany).

In all cases, a double scroll orientation was confirmed by direct visualization of the graft inside the injector under the microscope before insertion (Figure 2). In 4 of the cases, it was also confirmed with a microscope-integrated intraoperative OCT (OPMI LUMERA^®^ 700, ZEISS, Oberkochen, Germany; Figure 3). The injection of the roll was performed with a Geuder^®^ glass injector (Heidelberg, Germany), which was rotated in order to introduce the graft in the AC with the correct orientation. After this, the AC was shallowed immediately after graft insertion and prior to injector removal to maintain the orientation of the graft. Then, a 10/0 nylon single suture was placed in the main wound to seal the AC.

**Figure 4:**
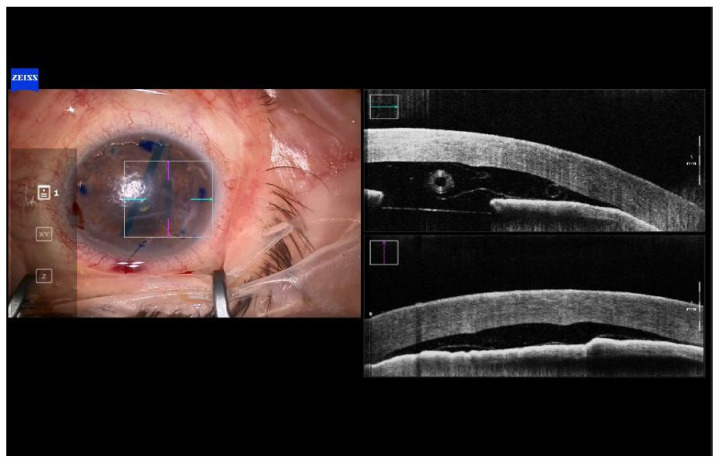
Case 3. Centering de graft and shallowing the AC before starting unscrolling maneuvers.

**Figure 5:**
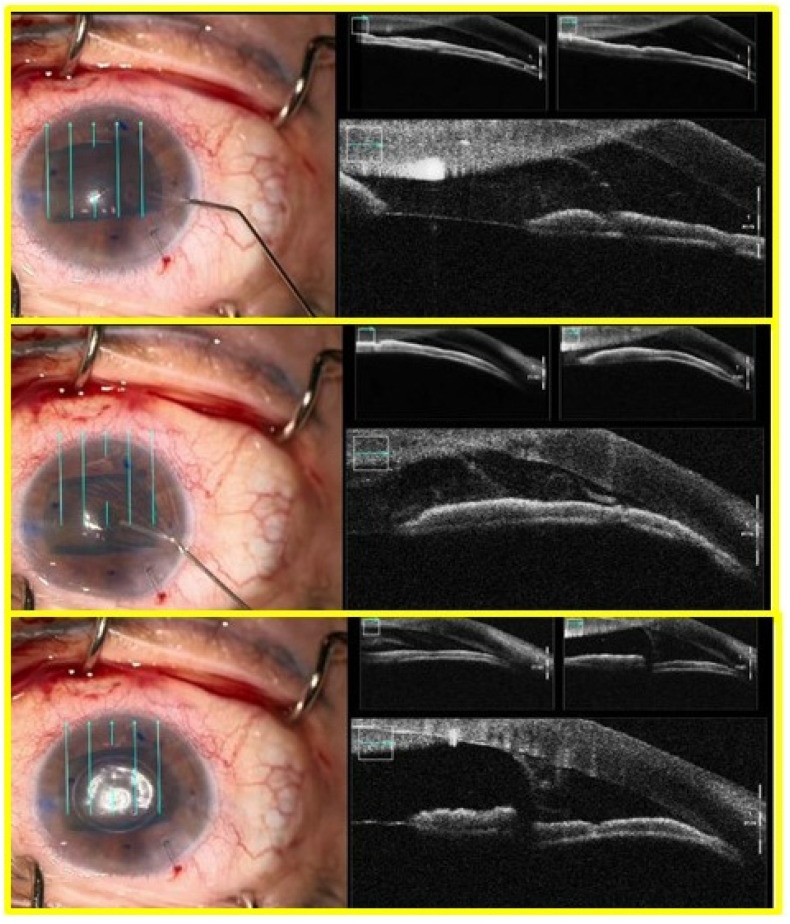
0.2 mL air bubble to fix centration and orientation of the graft before starting bubble bumping maneuvers.

**Figure 6:**
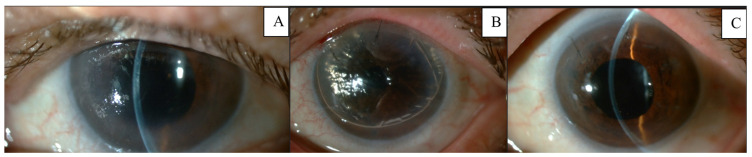
Case 2. Pre and postoperative images of one of our patients. (**A**) preoperative image with corneal oedema and Descemet folds. (**B**) postoperative image 24 h follow-up. (**C**) postoperative image 1-month follow-up.

Once the graft was inserted, the next step was performing centering maneuvers to ensure that the graft was properly positioned prior to unscrolling the tissue (Figure 4), which was achieved with tapping maneuvers on the graft. No BSS assistance should be used in these cases, as the AC must be kept shallow, to approximately 5 mm, to avoid rescrolling of the graft. When half of the double-roll was opened and centered, a 0.02 mL air bubble was injected underneath the graft with a 30 G Rycroft cannula in a 1 mL syringe (Figure 5). The air bubble helps hold the graft in place and prevents it from moving around during the procedure. Afterwards, the unscrolling was continued with the bubble-bumping technique, tapping on the anterior cornea and graft edges with a 25 G cannula. The AC was filled with sulfur hexafluoride (SF6) 20%. After 10 min, some gas was released until 80% of the AC was filled with SF6 gas. The patient was kept in a supine position for 2 h in the Ophthalmology ward before review and discharge. Patients were followed the next day, weekly the first month, and every 3 months up to 12 months (Figure 6).

## 3. Results

Five patients (three females and two males, mean age 71.2 ± 6.7 years) who underwent DMEK due to Fuchs Endothelial Cell Dystrophy (FECD) with no evidence of haze or posterior stromal fibrosis were included (Table 1). One of the patients had the DMEK surgery performed three months after a failed 4.5 mm DSO (Descemetorhexis Stripping Only). Another patient (case 1) had a shallow AC with an axial length of 18.4 mm. None of the patients were vitrectomized or had any other AC abnormalities.

Donors’ mean age was 23.6 ± 3.6 years (range 21 to 30), and the mean ECD was 2748.6 ± 162.6 cells/mm^2^ (Table 1). All of them underwent an uneventful DMEK as a single procedure performed by one experienced surgeon (MAG). The mean unfolding time was 7.2 ± 4.9 min (range 4 to 15).

Postoperative outcomes are collected in Table 2, including BCVA, ECD and CCT. BCVA at 6 months was 0.6 ± 0.2 with 1945.0 ± 455.5 cells/mm^2^ and 497.0 ± 19.7 microns of CCT. Cases 4 and 5 were performed 6 months ago, so data from the 12-month follow-up are not available (NA) yet. No cases of rebubbling or primary graft failure were observed. For up to 12 months, no cases of graft rejection or immune reaction have been noted. None of the patients had increased intraocular pressure, and macular OCT was normal in all follow-ups. No association between donor characteristics and the recipient outcome was noted.

## 4. Discussion

DMEK has become the superior form of endothelial keratoplasty in routine cases of corneal endothelial diseases in many countries worldwide. Although DMEK presents multiple advantages over DSAEK, such as less graft rejection and faster visual recovery, graft preparation and the learning curve are important limitations of DMEK. Successful DMEK surgery depends on a range of factors, including surgical skill, tissue processing technique and donor selection criteria. While younger donor corneas may pose a challenge during DMEK surgery due to tighter rolls, younger donors also tend to have higher ECD counts, which could thus improve transplant outcomes. Hence, it is essential to describe useful maneuvers in this type of donor grafts, which we have proven to have a strong tendency towards forming double rolls.

Several techniques for graft stripping have been described, including the “standard no-touch technique,” which was described in detail by Melles in 2008 and then further modified by hydrodissection or air dissection. It has previously been published that young donors have strong adherences, which may increase the risk of tears. However, there is no clear evidence in the literature that one of these techniques is more or less suitable for DMEK graft preparation in young donor corneas. In our case series, the graft preparation, starting at the scleral spur, was uneventful in all five cases with no tears or any other event [7,8].

Graft orientation during the surgery is the first critical step for a successful outcome, especially when using young donors and tight rolls. In all of our cases, the double scroll graft was visually inspected before insertion and set so that the scrolls were facing upwards. It is not that easy with older donors when a single roll is formed in the injector. For those cases, the Veldman Venn technique, which was first described by Peter Veldman, is very useful [9]. It is also important to confirm proper orientation after insertion. We have been using the intraoperative OCT for three years, which allows real-time high-resolution imaging during surgery and provides accurate and detailed information about graft placement, improving our surgical outcomes (Figure 1, Figure 2, Figure 3, Figure 4 and Figure 5). There are different options for that purpose, such as the Moutsouris sign, the Berrospi sign, an asymmetric letter or a peripheral mark [10]. Recently, Kobayashi et al. described a simple method for determining the graft orientation of tightly scrolled rolls by using endo-illumination [11]. In addition, Dapena et al. described the importance of the double-roll shape of the DMEK graft with endothelial cells facing downward (“external part”) prior to introducing the graft in the injector. This was considered an essential step, facilitating the unfolding maneuver and the subsequent steps [12].

When it comes to dealing with extreme young donors (e.g., under 30 years of age), despite being a “supertight” roll, we have hereby demonstrated that they tend to fold in a “double-roll” configuration, which is a remarkable and new finding. The unfolding of double-roll grafts is known to be easier and faster. In fact, Odkell et al. recently described a technique for forming a double-scroll graft from a single-scroll graft without causing additional damage to the graft [13]. Their technique consisted of controlled BSS bursts during graft preparation, and they reported a 70% success rate in less experienced technicians and a 90% success rate in experienced ones. Based on our experience, the following three key steps are essential to allow a fast and controlled unfolding of a very young donor: graft injection in the right orientation, shallow AC to 5 mm with no BSS assistance and a small central air bubble under the graft to fix the center to the host stroma. The air bubble is used to help hold the roll in place, centered and in the correct orientation.

To date, there are only a few papers published in the literature that have described DMEK with young donors (Table 3). None of the publications focus particularly on the surgical technique and tips to face the peculiarities and challenges with these grafts [14,15]. The unfolding is suggested to be more challenging due to the aforementioned characteristics of the DMEK grafts [16]. However, Maier et al. reported no correlation between donor age and the degree of difficulty of unfolding and attaching the endothelial grafts, although all donors were above 49 years of age and the mean age was 65 years [17]. As Vasanthananthan et al. described, there could be some advantages to having a tight roll. It could be easier to acquire the double-roll shape, facilitating its unfolding and the orientation check would be more obvious if the graft was upside-down [18]. Vasquez-Perez et al. described “the Spinning Technique” for unfolding tightly scrolled DMEK grafts, which involved injecting short bursts of BSS to make the scroll spin continuously in a 360-degree manner. Spinning of the graft over the surface was accompanied by rotation on its horizontal axis, resulting in an opening in the correct orientation. However, their youngest donor in their short series was 37 years old [5].

The disparity of results regarding young donors has been described in the literature. Rodriguez-Calvo et al. reported more complications with young donor corneas (>38 years), including spontaneous free-floating DMEK grafts, graft detachments or rebubbling, which were statistically significant compared to older donors (*p* = 0.049) [19]. In their series, a retrospective analysis of 334 DMEK was performed, but the mean donor age was 65 years (range, 38–85 years), and it remains unclear how many younger donors were included. In 2016, Schaub et al. concluded that the use of donor corneas ≤ 55 years (down to 17 years) for DMEK surgery is safe, and when the use of these grafts is limited to eyes with normal or shallow AC without previous vitrectomy, clinical outcome and complication rates are similar to those of older donor corneas [2]. According to Hill et al., the use of younger donors (younger than 50 years) did not result in higher rates of rebubbling or higher rates of ECD loss in the hands of the experienced surgeons, though they did not include donors as young as those included in our study [20].

There are other challenging patient’s and graft’s characteristics we can encounter when doing DMEK surgery, such as severe corneal edema, posterior stromal fibrosis, small eyes, big grafts, very deep or shallow AC, release of fibrin in AC if strong tapping, moderate manipulation or previous phacoemulsification surgery, dark irises, high vitreous pressure or no vitreous pressure in vitrectomized eyes. Many authors around the world have described several techniques or ways to overcome these limitations, including debriding epithelium to improve visibility and endo-illumination to see the edges of the graft if there is poor visualization [14]. We do not recommend using very young donors in such cases unless you have the technical support of an intraoperative OCT. In our series, FECD dystrophy patients who were not good candidates for DSO and had no challenging characteristics such as previous vitrectomy, deep AC or possible bad visualization were selected. In any case, it should be considered that achieving the learning curve of DMEK surgery, particularly for young donors, would be even more difficult.

Older donors (over 55 years old) are normally preferred as they are looser and unfold easier in DMEK surgery. Young donors’ corneas are not commonly selected due to the tight-roll behaviour and the longer unfolding times [21]. However, there is approximately a 35% cell loss due to graft preparation and DMEK surgery, which makes young donor corneas desirable because of their high ECD [17,19]. The slight loss of endothelial cells in our cases, comparing the eye bank information with the postoperative exam, suggested that with this technique, the manipulation was low, and the outcomes were satisfying. Therefore, the decision to exclude young donors from becoming DMEK tissue donors should be made with caution, taking into consideration not only the rolling behaviour of the donor tissue but also ECD and the recipient’s characteristics. Each eye bank and surgeon may have their own criteria for selecting donor corneas based on their experience and expertise in performing DMEK surgery. Ultimately, the goal is to overcome the shortage of old donors with high ECD and maximize the chances of a successful outcome for the patient.

In summary, we hereby present possible keys to overcome the tightly scrolled grafts of very young donors. These corneas are perfectly suitable for DMEK surgery without technical difficulties peeling off the graft. The graft shape tends towards a double-roll, and it is important to ensure the correct orientation when injecting into the AC. Shallowing the AC and placing a small bubble between the graft and the iris are strongly recommended maneuvers. Therefore, although most surgeons may prefer using corneas from older donors for DMEK because younger donors tend to have tighter rolls that may be more challenging to unfold, proper training, and technique refinement and instrumentation may help unfold tight young donor grafts and improve patients’ outcomes.

## Figures and Tables

**Table 1:** Donor and patients’ characteristics.

	Donor’s Age (Years)	Donor’s ECD (Cells/mm^2^)	Preservation Time (Days)	Size (mm)	Recipient’s Age (Years)	Sex	Unfolding Time (Minutes)
Case 1	22	2567	3	8.25	72	F	15
Case 2	22	2698	4	8	62	M	4
Case 3	21	2719	4	8	80	F	4
Case 4	30	3012	3	8	68	M	9
Case 5	23	2747	4	8	74	F	4

ECD (endothelial cell density), F (Female), M (Male).

**Table 2:** Postoperative data.

	BCVA			ECD		CCT		
	Preoperative	6 Months	12 Months	6 Months	12 Months	Preoperative	6 Months	12 Months
Case 1	CF	0.3	0.3	1267	1163	789	493	532
Case 2	0.2	0.8	0.9	1919	1807	890	524	540
Case 3	CF	0.7	0.7	1865	1801	768	469	497
Case 4	0.3	0.9	NA	2504	NA	698	497	NA
Case 5	0.1	0.5	NA	2170	NA	702	502	NA

CF (counting fingers), BCVA (best-corrected visual acuity), ECD (endothelial cell density), CCT (central corneal thickness), NA (not available).

**Table 3:** Similar publications with young donors.

Author	Year Published	Journal	Youngest Donor Age
Heinzelmann S	2014	*Cornea*	1 was 34 y.o, 2 > 40 y.o
Gorovoy IR	2014	*Cornea*	49 y.o
Maier AK	2015	*Graefes Arch. Clin. Exp. Ophthalmol.*	≥49 y.o
Sales CS	2016	*Cornea*	Only 3 donors < 55 y.o
Rodriguez-Calvo de Mora M	2016	*JAMA Ophthalmol.*	38 y.o
Schaub F	2016	*Am. J. Ophthalmol.*	Youngest group 17–40 y.o
Hill J	2021	*Cornea*	40 y.o.
Vasquez-Perez A	2022	*Cornea*	37 y.o

y.o: years old.

## Data Availability

Data available within the article or its Supplementary Materials.

## Supplementary Materials

The following supporting information can be downloaded at: https://www.mdpi.com/article/10.3390/jcm12196316/s1, Video S1: Surgical technique.
